# Nationwide analysis of olfactory neuroblastoma in Japan: evolving treatment approaches and prognostic outcomes

**DOI:** 10.1093/jjco/hyaf204

**Published:** 2025-12-26

**Authors:** Mariko Sekimizu, Takuya Mikoshiba, Ryoto Nagai, Naoaki Ishikawa, Takashi Okada, Megumi Kitayama, Daisuke Kawakita, Ken-ichi Nibu, Seiichi Yoshimoto, Hiroyuki Ozawa

**Affiliations:** Department of Otolaryngology, Head and Neck Surgery, Keio University School of Medicine, 35 Shinanomachi, Shinjuku-ku, Tokyo 160-8582, Japan; Department of Otolaryngology, Head and Neck Surgery, Keio University School of Medicine, 35 Shinanomachi, Shinjuku-ku, Tokyo 160-8582, Japan; Department of Otolaryngology, Head and Neck Surgery, Keio University School of Medicine, 35 Shinanomachi, Shinjuku-ku, Tokyo 160-8582, Japan; Department of Otolaryngology, Head and Neck Surgery, Keio University School of Medicine, 35 Shinanomachi, Shinjuku-ku, Tokyo 160-8582, Japan; Department of Otolaryngology, Head and Neck Surgery, Keio University School of Medicine, 35 Shinanomachi, Shinjuku-ku, Tokyo 160-8582, Japan; Data Center Department, Clinical Study Support Center, Wakayama Medical University Hospital, 811-1 Kimiidera, Wakayama-shi, Wakayama 641-8510, Japan; Japan Society for Head and Neck Cancer, 2-4-11 Fukagawa, Koto-ku, Tokyo 135-0033, Japan; Japan Society for Head and Neck Cancer, 2-4-11 Fukagawa, Koto-ku, Tokyo 135-0033, Japan; Department of Otorhinolaryngology, Head and Neck Surgery, Nagoya City University Graduate School of Medical Sciences, 1 Kawasumi, Mizuho-cho, Mizuho-ku, Nagoya, Aichi 467-8601, Japan; Japan Society for Head and Neck Cancer, 2-4-11 Fukagawa, Koto-ku, Tokyo 135-0033, Japan; Department of Otolaryngology, Kobe University School of Medicine, 7-5-1 Kusunoki-cho, Chuo-ku, Kobe, Hyogo 650-0017, Japan; Japan Society for Head and Neck Cancer, 2-4-11 Fukagawa, Koto-ku, Tokyo 135-0033, Japan; Department of Head and Neck Surgery, National Cancer Center Hospital, 5-1-1 Tsukiji, Chuo-ku, Tokyo 104-0045, Japan; Department of Otolaryngology, Head and Neck Surgery, Keio University School of Medicine, 35 Shinanomachi, Shinjuku-ku, Tokyo 160-8582, Japan

**Keywords:** olfactory neuroblastoma, endoscopic surgery, radiotherapy, recurrence-free survival, nationwide registry

## Abstract

**Background:**

Olfactory neuroblastoma (ONB) is a rare malignant tumor of the nasal cavity and paranasal sinuses. In this study, we aimed to analyze ONB cases registered in the nationwide Head and Neck Cancer Registry of Japan.

**Methods:**

Among 90 885 head and neck cancer registrations (2011–19), we identified 346 patients with ONB. We summarized demographics, tumor-node-metastasis (TNM) classification, and treatment modalities (surgery, radiotherapy, chemotherapy) and compared patterns between an early (2011–15) and a late (2016–19) period. Survival was analyzed in 95 patients with standardized 5-year outcomes available.

**Results:**

T4 lesions were frequent, and 234 patients (67.6%) received surgery-based treatment, typically combined with postoperative radiotherapy. Over time, endoscopic approaches increased markedly and became predominant over open skull base surgery. Among the 95 patients with evaluable follow-up, the 5-year overall survival (OS) and 5-year recurrence-free survival (RFS) were 85.1% and 62.7%, respectively. Patients <60 years old and female patients exhibited better OS compared to younger patients and males. Postoperative radiotherapy was associated with improved RFS but not OS. Chemotherapy was used more often with open skull base surgery than with other surgical approaches.

**Conclusions:**

Endoscopic surgery for ONB rose substantially, while younger age and female sex were associated with better OS, and postoperative radiotherapy was correlated with improved RFS.

## Teaser

Analyzed nationwide data on olfactory neuroblastoma in Japan, highlighting increased endoscopic surgery, survival differences by age and sex, and improved recurrence-free survival with postoperative radiotherapy.

## Introduction

Olfactory neuroblastoma (ONB) is a rare malignant tumor arising from the olfactory epithelium; it accounts for ~3%–6% of malignancies of the nasal cavity and paranasal sinuses [1]. The current standard treatment is surgical resection followed by postoperative radiotherapy. Historically, open anterior skull base surgery via frontal craniotomy was the predominant approach for anterior skull base tumors. With the increasing adoption of endoscopic endonasal skull base surgery, ONB surgical management has become less invasive. Conversely, despite high short-term local control rates with surgery plus postoperative radiotherapy, long-term outcomes remain unsatisfactory, with a 10-year disease-free survival of around 51%–62% [2–4], indicating an increasing cumulative risk of recurrence over time. Moreover, although endoscopic techniques are now widely used, consistent improvements in survival have not been demonstrated [4]. These observations highlight that improving ONB outcomes will require optimizing treatment strategies, guided by evidence from large-scale clinical data.

Because ONB is rare, most reports originate from single institutions with only dozens of patients, limiting robust characterization of real-world treatment patterns and outcomes, underscoring the need for large-scale, multi-institutional, registry-based analyses requiring a nationwide database. The Head and Neck Cancer Registry of Japan [5], maintained by the Japan Society for Head and Neck Cancer, aggregates annual case registrations from most institutions treating head and neck malignancies across Japan and includes a standardized 5-year outcome survey initiated in 2017.

Using this nationwide registry, we described ONB national treatment patterns and temporal trends. In a subset with standardized 5-year outcome data, we further evaluated factors associated with overall and recurrence-free survival.

## Patients and methods

**Figure 1:**
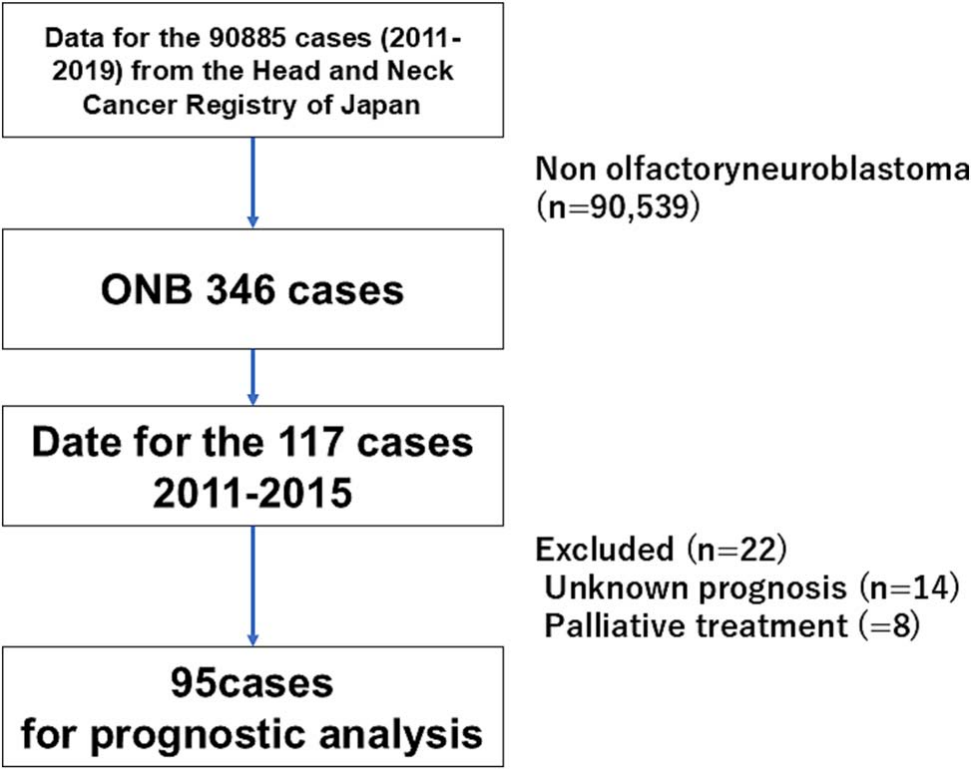
STROBE flow diagram of this observational study.

We analyzed patients diagnosed and registered between 2011 and 2019. Of 90 885 head and neck cancer cases in this registration period, 346 exhibited ONB (Fig. 1). Patient demographics and initial treatments were summarized for all 346 cases, and period-based comparisons were performed between an early (2011–15; *n* = 117) and a late (2016–19; *n* = 229) period. This division into two periods was due to the addition of the “endoscopic skull base surgery” procedure category starting in 2016; before that, “endoscopic resection” was the available option for this procedure. The larger case number in the late period primarily reflected increased institutional participation in the registration over time.

The modified Kadish staging [6] and Dulguerov staging [7] are commonly used for ONB staging, whereas the Hyams classification describes histologic grade; none were registry input items. Therefore, tumor extent was therefore recorded using the conventional TNM classification for sinonasal cancers; T4 encompassed T4a and T4b because the registry did not distinguish between them. Because no validated algorithm converts TNM to Dulguerov or Kadish staging, we did not attempt any cross-classification. For analysis, surgical procedures were grouped as “endoscopic skull base surgery,” “open skull base surgery,” “maxillectomy,” or “other and unknown.” Surgical input options are listed in Supplementary Table S1.

For survival analyses, we used cases registered in 2011–15 with available 5-year outcomes; events beyond 5 years were not systematically collected, although follow-up could be solicited *ad hoc* from institutions. Among 117 ONB cases in that period, 8 received palliative treatment only and 14 lacked prognostic data; thus, 95 patients comprised the final survival cohort. Overall survival (OS) and recurrence-free survival (RFS) were estimated using Kaplan–Meier methods with two-sided log-rank tests for between-group comparisons. Comparisons between the early and late periods used the Mann–Whitney U test for age and Pearson’s χ^2^ test for categorical variables, with Fisher’s exact test when expected cell counts were small; unknown categories were excluded. As a sensitivity analysis, treatment sequences were evaluated using both the full categorization and a prespecified aggregated categorization. A two-sided *P*-value <.05 was considered statistically significant. Analyses were conducted with IBM SPSS Statistics (version 29.0; IBM Corp., Armonk, NY, USA).

## Results

### Analysis of all registered cases

Tables 1 and 2 summarize the patient demographics. Overall, 208 patients (60.1%) were male, and 138 (39.9%) were female, with a median age of 55 years (range 0–88). The primary tumor sites were as follows: nasal cavity, ethmoid sinus, maxillary sinus, sphenoid sinus, posterior nasal aperture (including posterior end of the nasal septum), and nasal sinus not otherwise specified in 209 (60.4%), 40 (11.6%), 5 (1.4%), 3 (0.8%), 3 (0.8%), and 86 (24.9%), respectively. Regarding T classification, T1, T2, T3, and T4 diseases were identified in 21 (6.1%), 46 (13.3%), 21 (6.1%), and 118 (34.1%) patients, respectively, with 140 (40.5%) unspecified cases. For N classification, 214 (61.8%), 21 (6.1%), and 111 (32.1%) were N0, N1-3, and unspecified, respectively. Three patients (0.9%) presented with distant metastases (M1) at diagnosis (one with lung metastasis and two with liver metastases).

**Table 1:** Patient characteristics

Variable	All patients (*n* = 346)		Early period (*n* = 117)		Late period (*n* = 229)		*P-*value
	*n*	(%)	*n*	(%)	*n*	(%)	
**Age**	–	–	–	–	–	–	.115
Median (range)	55 (0–88)		59 (20–88)		54 (0–88)		–
**Sex**	–	–	–	–	–	–	.216
Male	208	60.1	65	–	143	–	–
Female	138	39.9	52	–	86	–	–
**Performance status**		–	–	–	–	–	.711
0	246	71.1	73	–	173	–	–
1	32	9.2	9	–	23	–	–
2	7	2.0	2	–	5	–	–
3	2	0.6	0	–	2	–	–
Unknown	59	17.1	33	–	26	–	–

*P*-values from the Mann–Whitney U test (age) and χ^2^ test (categorical variables). Percentages are column percentages; unknowns were excluded from tests. –, not applicable.

**Table 2:** Disease characteristics

Variable	All patients (*n* = 346)		Early period (*n* = 117)		Late period (*n* = 229)		*P-*value
	*n*	(%)	*n*	(%)	*n*	(%)	
**Primary site**	–	–	–	–	–	–	.393
Nasal cavity	209	60.4	67	57.3	142	62	–
Maxillary sinus	5	1.4	1	0.8	4	1.7	–
Ethmoid sinus	40	11.6	22	18.8	18	7.9	–
Sphenoid sinus	3	0.8	1	0.8	2	0.9	–
Posterior nasal aperture	3	0.8	0	0	3	1.3	–
NOS	86	24.9	26	22.2	60	26.2	–
**T classification**	–	–	–	–	–	–	.037
1	21	6.1	2	1.7	19	8.3	–
2	46	13.3	15	12.8	31	13.5	–
3	21	6.1	7	6.0	14	6.1	–
4	118	34.1	42	35.9	76	33.2	–
No definition/unknown	140	40.5	51	43.6	89	38.9	–
**N classification**	–	–	–	–	–	–	.954
0	214	61.8	70	59.8	144	62.9	–
1	9	2.6	2	1.7	7	3.1	–
2	11	3.2	5	4.3	6	2.6	–
	1	0.3	0	0	1	0.4	–
No definition/unknown	111	32.1	40	34.2	71	31	–
**M classification**	–	–	–	–	–	–	.225
0	249	72	83	70.9	166	72.5	–
1	3	0.8	2	1.7	1	0.4	–
No definition/unknown	94	21.2	32	27.4	62	27.1	–

Posterior nasal aperture included posterior end of the nasal septum, and NOS included nasal sinus not otherwise specified. T4 includes T4a and T4b (registry did not distinguish). *P*-values from χ^2^ test; unknown categories excluded from tests. –, not applicable.

Regarding treatment strategies, of all 346 patients, 301 (87.0%) were treated with radical intent, 20 (5.8%) received palliative treatment, and treatment information was unspecified for 25 (7.2%) cases (Table 3). Among patients treated with radical treatment, 234 (77.7%) underwent surgery with or without additional treatment: 52 (17.3%) had surgery alone, 161 (53.5%) had surgery followed by postoperative radiotherapy or chemoradiotherapy, 15 (5.0%) had surgery plus radiotherapy or chemoradiotherapy and chemotherapy, and 6 (2.0%) had surgery plus chemotherapy. Surgical procedures included endoscopic skull base surgery (*n* = 105), open skull base surgery (*n* = 51), maxillectomy (*n* = 24), and other or unknown procedures (*n* = 54).

**Table 3:** Treatment characteristics

Variable	All		Early period		Late period		*P-*value
**Treatment policy**	(*n* = 346)		(*n* = 117)		(*n* = 229)		1.000
	*n*	(%)	*n*	(%)	*n*	(%)	
Radical treatment	301	87	102	87.2	199	86.9	–
Palliative care	20	5.8	8	6.8	12	5.2	–
Unknown	25	7.2	7	6.0	18	7.9	–
**Treatment sequence** **(of radical treatment)**	(*n* = 301)		(*n* = 102)		(*n* = 199)		.034
	*n*	(%)	*n*	(%)	*n*	(%)	
Surgery alone	52	17.3	14	13.7	38	19.1	–
Surgery + RT or CRT	161	53.5	46	45.1	115	57.8	–
Surgery + RT or CRT + CTX	15	5.0	6	5.9	9	4.5	–
Surgery + CTX	6	2.0	5	4.9	1	0.5	–
RT alone	20	6.6	8	7.8	12	6.0	–
CRT alone	12	4.0	5	4.9	7	3.5	–
RT or CRT + CTX	24	8.0	13	12.7	11	5.5	–
CTX alone	7	2.3	2	2.0	5	2.5	–
Unknown	4	1.3	3	2.9	1	0.5	–
**Surgical procedure** **(only surgery performed)**	(*n* = 234)		(*n* = 71)		(*n* = 163)		<.0001
	*n*	(%)	*n*	(%)	*n*	(%)	
Endoscopic skull base surgery	105	44.9	3	4.2	102	62.6	–
Open skull base surgery	51	21.8	23	32.4	28	17.2	–
Maxillectomy	24	10.3	4	5.6	20	12.3	–
Other and unknown	54	23.1	41	57.7	13	8.0	–

Treatment sequence in no particular order. Percentages were calculated as column percentages among radical-treatment patients, with unknown values included in the denominator. Unknown categories were excluded from hypothesis testing. *P*-values: Pearson’s χ^2^ test (unknown excluded for sequence); Fisher’s exact test for 2 × 2 where applicable.

RT, radiotherapy or particle beam therapy; CRT, concurrent chemoradiotherapy; CTX, systemic chemotherapy; –, not applicable.

For non-surgical treatment, 56 (18.6%) patients received radiation-based therapy, comprising particle beam therapy in 31 patients and conventional photon (X-ray) radiotherapy in 25. Among these 56 patients, radiation alone, concurrent chemoradiotherapy, and radiation (or chemoradiation) combined with systemic chemotherapy were used in 20, 12, and 24 patients, respectively. Systemic chemotherapy alone was used as radical treatment in seven (2.3%) cases.

### Comparison between the early and late treatment periods

Patients enrolled in the early period (*n* = 117) were compared with those enrolled in the late period (*n* = 229 patients). The median ages for the early and late periods were 59 and 54 years, respectively. There were no notable differences in the distribution of performance status, primary site, and N classification. Nonetheless, for T classification, excluding patients with unknown T classification, the comparison between T1 and all other T categories (T2/3/4) reached statistical significance (*P* = .037), indicating a higher proportion of T1 lesions in the late period (Tables 1 and 2).

Radical treatment was performed in 102 (87.2%) and 199 patients (86.9%) patients during the early and late periods, respectively, with no significant difference between periods (Fisher’s exact *P* = 1.000; Table 3). Among patients treated with curative intent, the distribution of treatment sequences differed under the full categorization (χ^2^ = 15.19, df = 7, *P* = .0337; unknown excluded). Importantly, after excluding patients with unknown treatment sequence, the proportion receiving any surgery increased from 71/99 (71.7%) in the early period to 163/198 (82.3%) in the late period (Fisher’s exact *P* = .0496).

In patients who underwent surgery, the distribution of specific procedures differed markedly between periods (endoscopic skull base surgery/open skull base surgery/maxillectomy/other and unknown: χ^2^ = 97.99, df = 3, *P* < .0001). In the early period, 32.4% and 4.2% of cases underwent open skull base surgery and endoscopic skull base surgery, respectively; in the late period, open skull base surgery and endoscopic skull base surgery decreased and increased to 17.2% and 62.6%, respectively (Fisher’s exact for endoscopic vs open, *P* < .0001), indicating a clear shift toward endoscopic approaches over time. Although the “Other” category of the registry may have limited the precision of surgical classification, the direction and magnitude of this shift were consistent across tests.

### Prognostic analysis for 95 cases

Patient characteristics and treatment details are presented in Table 4. Among the 95 included patients, 54 (56.8%) were male, and 41 (43.2%) were female, with a median age of 60 years (range 20–88). T4 disease was the most common (37 patients, 38.9%), while T-stage information was unavailable for 35 patients (36.8%). Sixty-three (66.3%) and six (6.3%) patients were N0 and N1–N2, respectively; N-classification was unavailable for 26 (27.4%).

**Table 4:** Patient characteristics of 95 cases for prognostic analysis

Variable	*n*	(%)
**Age (years)**	–	–
Median (range)	60 (20–88)	–
**Sex**	–	–
Male/female	54/41	56.8/43.2
**T classification**	–	–
1/2/3	2/14/7	2.1/14.7/7.4
4(4, 4a, 4b)	37	38.9
No definition/unknown	35	36.8
**N classification**	–	–
0	63	66.3
1/2	1/5	1.1/5.3
No definition/unknown	26	27.4
**Treatment sequence(of radical treatment)**		
Surgery alone	13	13.7
Surgery + RT/CRT	40	42.1
Surgery + RT + CTX	8	8.4
Surgery + CTX	3	3.2
RT alone	7	7.4
CRT alone	5	5.3
RT/CRT + CTX	13	13.7
CTX alone	2	2.1
Unknown	4	4.2

Ninety-five cases registered during 2011–15 with a 5-year outcome survey. Unknown stage categories were excluded from survival comparisons. Categories are mutually exclusive.

RT, radiotherapy; CRT, concurrent chemoradiotherapy; CTX, chemotherapy; –, not applicable.

Regarding treatment, 64 patients underwent surgery-based treatment (surgery with or without perioperative radiotherapy and/or systemic chemotherapy), 25 received radiation-based therapy (particle beam therapy in 15 and X-ray therapy in 10), with or without chemotherapy, and 6 received systemic chemotherapy alone with curative intent. In the surgery-based treatment group, systemic chemotherapy was more common with open skull base surgery than with other surgical approaches (7/22 [31.8%] vs 4/42 [9.5%]; Fisher’s exact *P* = .02; Supplementary Table S2).

**Figure 2:**
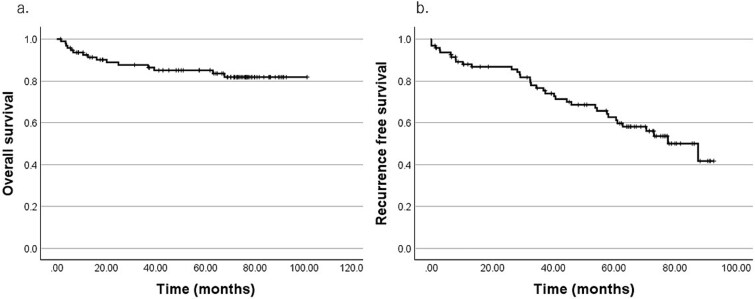
Kaplan–Meier survival curves of 95 patients. (a) Overall survival, (b) recurrence-free survival.

Of the 95 patients analyzed, 34 (35.8%) exhibited recurrence: 16 local recurrences at the primary site, 15 regional (cervical) lymph node metastases, and 5 distant metastases; categories were not mutually exclusive. The median follow-up period among survivors was 68.8 months (range, 0–92.8). At the 5-year follow-up, 74 patients were alive, 15 had died, and 6 were lost to follow-up. Moreover, the 5-year overall survival (5y-OS) and the 5-year recurrence-free survival (5y-RFS) rate were 85.1% and 62.7%, respectively (Fig. 2).

Univariate analyses by patient characteristics and treatment modalities (log-rank tests) are presented in Table 5. OS was significantly better in patients <60 years than in those ≥60 years (5y-OS, 95.3% vs 75.5%; *P* = .015). Additionally, female patients demonstrated significantly better OS than male patients (5y-OS, 94.7% vs 77.5%; *P* = .011). Although advanced disease (T4, N1–3) demonstrated numerically worse OS and RFS, these differences were not statistically significant.

**Table 5:** Univariate analysis by patient characteristics of 95 cases for prognostic analysis

	*n*	5y-OS (%)	*P*-value	5y-RFS (%)	*P-*value
**All patients**	95	85.1	–	62.7	–
**Age**	–	–	–	–	–
<60	46	95.3	.015	65.6	.936
≥60	49	75.5	–	59.9	–
**Sex**	–	–	–	–	–
Female	41	94.7	.011	60.6	.183
Male	54	77.5	–	65.5	–
**T classification**	–	–	–	–	–
T1/2/3	23	86.4	.484	69.1	.093
T4	37	85.6	–	47.2	–
**N classification**	–	–	–	–	–
N0	63	85.8	.248	57.6	.746
N1/2/3	6	83.3	–	50.0	–

*P*-values from log-rank tests.

5y-OS, 5-year overall survival; 5y-RFS, 5-year recurrence free survival; –, not applicable.

**Figure 3:**
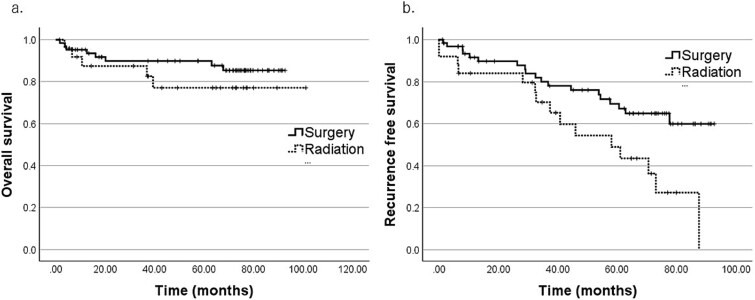
Primary treatment and overall survival (OS). Kaplan–Meier curves comparing surgery-based and radiation-based groups.

**Figure 4:**
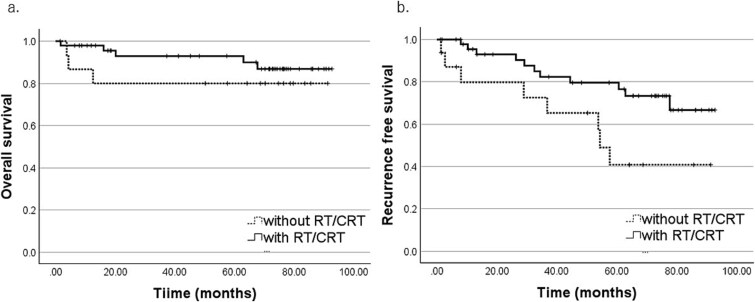
Primary treatment and recurrence-free survival (RFS). Kaplan–Meier curves comparing surgery alone vs surgery plus postoperative radiotherapy.

Regarding primary treatment (Table 6 and Fig. 3), RFS was significantly better in the surgery-based group than in the radiation-based group (5y-RFS, 69.4% vs 48.9%; *P* = .010). Within the surgery-based group, surgery plus radiotherapy yielded better RFS than surgery alone (5y-RFS, 79.6% vs 40.8%; *P* = .030) (Fig. 4). Although OS and RFS tended to be higher in patients who received systemic chemotherapy as part of curative treatment, these differences were not statistically significant.

**Table 6:** Univariate analysis by primary treatment of 95 cases for prognostic analysis

	*n*	5y-OS (%)	*P*-value	5y-RFS (%)	*P-*value
**Main treatment**	–	–	–	–	–
Surgery-based	64	89.9	.322	69.4	.010
Radiation-based	25	77.1	–	48.9	–
**Postoperative adjuvant therapy**			–	–	–
With RT/CRT	48	92.9	.36	79.6	.030
Without RT/CRT	16	80.0	–	40.8	–
**Addition of CTX**		–	–	–	–
With CTX	11	100.0	.658	80.0	.659
Without CTX	53	87.7	–	67.0	–

Log-rank test; strata as shown.

5y-OS, 5-year overall survival; 5y-RFS, 5-year recurrence free survival; RT, radiotherapy; CRT, concurrent chemoradiotherapy; CTX, chemotherapy; –, not applicable.

## Discussion

This study provides a nationwide, registry-based observational analysis of the characteristics of patients with ONB in Japan. Consistent with the findings of prior reports, ONB was slightly more common in males, than in females, with incidence peaking in the fifth to sixth decades [8–10]. The primary ONB site is typically the olfactory cleft or upper nasal cavity, although ectopic occurrences have also been reported [11, 12]. This study demonstrated that ONB was frequently diagnosed at an advanced T category (T4). Overall, 118/346 (34.1%) were classified as T4. Since the T category was unknown for 140 cases, the T4 proportion rose to 57.3% (118/206) when considering only those specified T staging. This finding aligns with those of previous large case series reporting that approximately half of ONB cases fall into Kadish C or Dulguerov T4 categories [12, 13], although a direct one-to-one mapping between TNM and these systems is not available. When comparing the early and late registration periods, the early-stage ONB proportion increased in the late period, potentially reflecting earlier tumor detection facilitated by wider use of flexible nasoendoscopy and improved access to cross-sectional imaging in Japan. While some site and stage distributions in our cohort were broadly comparable to those of prior series, others differed, likely reflecting cohort composition and registry definitions; hence, direct one-to-one concordance with previous reports should be interpreted cautiously.

In earlier practice, tumor excision commonly used an open anterior skull base approach via frontal craniotomy. Over the past two decades, with the introduction of endoscopic techniques, transnasal endoscopic skull base surgery has been widely adopted for ONB owing to its safety and favorable outcomes [14, 15]. Our study period coincided with this transition in Japan, shifting from routine open skull base surgery to a preference for endoscopic approaches. To evaluate this shift, we divided the period into early and late phases and observed a higher frequency of endoscopic surgery in the latter. Among patients treated with curative intent, the proportion receiving surgery-based treatment similarly increased, while radiation-based therapy decreased. This pattern aligns with the broader dissemination of endoscopic surgery in Japan and may reflect a move toward more active surgical selection. Although endoscopic surgery reduces invasiveness, some studies report no improvement in prognosis [4]. Despite the lack of clear survival enhancement, comparable oncologic outcomes with less invasiveness represent an important clinical advantage. In any case, improving long-term outcomes may require optimizing primary treatment and refining indications for perioperative adjuvant strategies, particularly postoperative regimens.

Here, 5y-OS and 5y-RFS rates were 85.1% and 62.7%, respectively, which were generally consistent with those of previous reports. ONB prognosis varies across studies, with 5y-OS rates ranging from 63% to 89% [10, 12, 16]. Although ONB is often considered to have a relatively favorable prognosis among sinonasal malignancies [17], late recurrence and metastasis remain important clinical concerns. Previous large series reported 5y-OS of 83%–86% and 10-year OS of 63%–76% [12, 16], comparable to our findings. Thus, while the short-term ONB prognosis is favorable, the risk of recurrence and subsequent mortality increases over time; improving long-term prognosis may require optimization of primary treatment and development of risk-adapted perioperative strategies, including postoperative adjuvant therapy.

Several ONB prognosis factors have been previously reported, particularly from population-based registries in the USA. These studies identified age, sex, Kadish stage, Hyams classification, lymph node metastasis, and margin status as key prognostic indicators [13]. In this study, 5y-OS was higher in patients <60 years and in female patients. Prior evidence on age is conflicting: some studies align with our finding of poorer outcomes in older adults [8], whereas others report greater aggressiveness in younger patients [18], possibly reflecting comorbidities and lower treatment tolerance in older individuals.

Sex effects are similarly inconclusive. Some studies show worse outcomes in men [8], while others attribute this to more advanced presentation [19, 20]. In our cohort, T and N classifications did not differ by sex, suggesting that histological grade or treatment strategy may be more influential; further research is warranted.

For ONB treatment, the combination of surgery and postoperative radiotherapy is widespread, as it improves local control and prolongs survival [1, 21, 22]. However, some reports have demonstrated improved local control without a corresponding survival benefit or have suggested that postoperative radiotherapy should be reserved only for locally advanced cases [12, 23]. In this study, postoperative radiotherapy improved RFS but did not significantly influence OS (e.g. 5y-RFS was higher in the irradiated group), potentially reflecting limited sample size, observation window, and missing data. Additionally, T classification and nodal status were not significant prognostic factors in univariate analyses. The registry implements a standardized 5-year outcome survey; beyond 5 years, outcomes were obtained on an *ad hoc* basis rather than systematically, limiting robust long-term inference.

Optimizing and strengthening perioperative treatment holds potential for ONB prognosis improvement. Although not statistically significant, OS and RFS tended to be higher in patients who received systemic chemotherapy as part of curative-intent treatment than in those who did not. In our cohort, chemotherapy was used more frequently with open skull base surgery than with other surgical approaches (31.8% vs 9.5%; *P* = .02), suggesting that its use often reflected more advanced tumor extent. Notably, platinum-based regimens (e.g. cisplatin/carboplatin-based combinations) are commonly used. The role of chemotherapy in ONB remains unclear [24]; nonetheless, some studies suggest potential benefit in locally advanced or unresectable disease [25, 26]. While some reports indicate that adding chemotherapy to radiotherapy improves prognosis [27, 28], systematic reviews have not demonstrated a consistent survival benefit [24]. Prospective, multicenter studies are necessary to evaluate the efficacy of chemotherapeutic agents, including molecularly targeted therapies [29, 30] and immune checkpoint inhibitors [31]. Given the rarity of ONB, demonstrating the effectiveness of such treatments will likely require basket or platform trials focusing on predefined molecular alterations and immune markers (e.g. vascular endothelial growth factor (VEGF) and programmed death-ligand 1 (PD-L1)).

This study has some limitations. First, because ONB is rare, even a nationwide registry yields modest sample sizes for subgroup analyses. The registry implements a standardized 5-year outcome survey; therefore, outcomes beyond 5 years were not systematically collected and, when reported, were obtained *ad hoc*, limiting robust long-term inference. Second, the registry lacks ONB-specific fields (e.g. Kadish stage, Dulguerov stage, and Hyams grade) and margin status. A direct one-to-one mapping between TNM and Dulguerov is not available; while both describe local extent, any conceptual correspondence should be interpreted cautiously. Third, surgical taxonomy reflects the era in which the registry was established: combined endoscopic surgery with open craniotomy was not assumed and may have been classified as open skull base surgery. Furthermore, the 2016 addition of “endoscopic skull base surgery” as a registry option introduces potential registration bias in temporal comparisons. Furthermore, inter-institutional heterogeneity in treatment selection and variability in data entry may have introduced unmeasured confounding and misclassification. Additionally, missing data raises concerns regarding treatment and survival estimate precision. Finally, as an observational study, our analyses demonstrate associations rather than causation; prospective, stage-adjusted multicenter studies, ideally including clinical trials where feasible, are warranted to define prognostic factors and evaluate perioperative and postoperative adjuvant strategies in ONB.

## Conclusions

Over the study period in Japan, endoscopic skull base surgery markedly increased and coincided with a shift toward surgery-based management of ONB. Among evaluable patients, younger age and female sex were associated with superior OS, while postoperative radiotherapy correlated with improved RFS. These findings, while observational, suggest that advances in endoscopic techniques may have broadened operability in clinical practice. Future work should define selection criteria and postoperative strategies that translate procedural advances into durable survival gains.

## Supplementary Material

Supplemental_Tables_hyaf204

## Data Availability

The data generated in this study are available in the article.
