# Supplementary material for: Nationwide analysis of olfactory neuroblastoma in Japan: evolving treatment approaches and prognostic outcomes
Source: Jpn J Clin Oncol. 2025 Dec 26;56(3):282–9. doi: 10.1093/jjco/hyaf204 (PMC13017732; doi:10.1093/jjco/hyaf204)
Supplement: Supplemental_Tables_hyaf204 [file supplemental_tables_hyaf204.docx]

Supplemental Table 1. Registry input options for surgical procedures.

| **Summarized the surgical procedure in this study** | **Input option of surgical procedure in the registry** |
| --- | --- |
| Endoscopic skull base surgery | Endoscopic skull base surgery (from 2016)  Endoscopic resection |
| Open skull base surgery | Skull base dissection  Extensive skull base tumor resection  Anterior skull base resection  Other resections involving the anterior skull base |
| Maxillectomy | Partial maxillectomy  Total maxillectomy  Other types of maxillectomy |
| Other and Unknown | Others  Unknown  Blank |

Supplemental Table 2. Surgical approaches among surgery cases by perioperative systemic chemotherapy status (n=64).

| Surgical procedure | All  (n=64) | | With CTX  (n=11) | | Without CTX  (n=53) | | *p-*value* |  |
| --- | --- | --- | --- | --- | --- | --- | --- | --- |
|  | n | (%) | n | (%) | n | (%) |  | |
| Endoscopic skull base surgery | 2 | 3.1 | 0 | 0 | 2 | 3.8 |  | |
| Open skull base surgery | 22 | 34.4 | 7 | 63.6 | 15 | 28.3 |  | |
| Maxillectomy | 4 | 6.2 | 0 | 0 | 4 | 7.5 |  | |
| Other and Unknown | 36 | 56.3 | 4 | 36.4 | 32 | 60.4 |  | |
| Open skull base surgery | 22 | 34.4 | 7 | 63.6 | 15 | 28.3 | 0.02 | |
| Non-open surgery | 42 | 65.6 | 4 | 36.4 | 38 | 71.7 |  | |

CTX: Chemotherapy. Percentages are column percentages; categories are mutually exclusive. * *p-*value from a two-sided Fisher’s exact test for Open vs Non-open (Endoscopic + Maxillectomy + Other/Unknown).
